# High-Precision Two-Dimensional Angular Sensor Based on Talbot Effect

**DOI:** 10.3390/s24227333

**Published:** 2024-11-17

**Authors:** Liuxing Song, Xiaoyong Wang, Jinping He, Guoliang Tian, Kailun Zhao

**Affiliations:** 1 College of Astronautics, Nanjing University of Aeronautics and Astronautics, Nanjing 211106, China; 2 Beijing Institute of Space Mechanics and Electricity, Beijing 100081, China

**Keywords:** angular detection, Talbot effect, optical alignment, 3D printing technology

## Abstract

The precision of two-dimensional angular sensing is crucial for applications such as navigation, robotics, and optical alignment. Conventional methods often struggle to balance precision, dynamic range, and affordability. We introduce a novel method leveraging the Talbot effect, enhanced by 3D printing technology, to fabricate a grating prototype for high-precision angular measurements. The method detects amplitude grating displacement at the self-imaging position and employs a frequency filtering algorithm for improved accuracy. Rigorous validation through simulations and physical experiments demonstrates that our method achieves a detection resolution of 0.4 arcseconds and a dynamic range exceeding 1400 arcseconds. This research presents a cost-effective, high-precision angular detection solution with potential for broad application in precision mechanical assembly, optical alignment, and other relevant domains.

## 1. Introduction

In contemporary precision optical systems, the accuracy of angle detection is paramount for ensuring system performance, particularly in fields such as optical imaging, adaptive optics, and laser communication [1,2]. Tip–tilt errors, which are deviations from the desired alignment, can significantly degrade image quality and signal stability. Precise measurement and correction of these tilt errors are essential for the realization of high-performance optical systems [3]. The existing methods for measuring tilt errors are primarily optoelectronic, which offer unique advantages and challenges related to cost, complexity, and environmental sensitivity [4,5].

Optoelectronic methods are differentiated into geometric optics-based and wave optics-based techniques. Geometric optics methods, such as autocollimators, are well-suited for a variety of high-precision optical measurements due to their ability to calculate angle deviations by measuring the offset of the focal point image. However, autocollimators necessitate complex optical lens assemblies, which contribute to large system volumes, high costs, and limited portability [6,7,8]. Wave optics-based methods encompass interferometric and moiré fringe techniques. Interferometric methods capitalize on the interference of beams to detect minute angle changes through phase differences, offering unrivaled precision and sensitivity for minute angle variations [9]. Despite their high precision, these methods are constrained by a limited dynamic range and susceptibility to environmental perturbations, such as vibrations and temperature fluctuations. Moiré fringe methods, fundamentally rooted in the Talbot effect, have seen significant advancements in recent years that address some of the aforementioned issues.

Recent studies have demonstrated the potential of moiré fringe techniques in high-precision angular measurement. Fan et al. introduced a method for aligning two gratings, showcasing the potential for angle measurement [10]. J. Romijn et al. utilized a dual-grating system to convert angular information into intensity information, while Meng et al. further advanced this approach by employing on-chip circuitry for angle detection above CMOS structures. These methods offer a wide dynamic range but are limited by lower precision [11,12]. Li et al. improved detection accuracy significantly by employing a dual-grating and lens configuration, though it requires precise alignment of the two gratings [13]. Yang investigated the impact of grating period and other parameters on angle detection, but the focus of his detection was on the relative angular changes between gratings [14]. Suzuki et al. proposed a single-grating and lens approach, which still necessitates the construction of a complex optical setup [15]. Lastly, Xin et al. introduced a method employing diffractive optical elements. Despite its novel approach, the method has not yet reached high precision levels [16].

In response to the traditional methods’ shortcomings, such as imprecision, narrow dynamic ranges, and the high costs associated with their implementation, this paper presents an innovative angle detection method based on the Talbot effect. This innovative approach calculates angle deviations from the near-field diffraction patterns of periodic gratings, streamlining system architecture. The method is distinguished by its minimalist requirement for a single-layer grating, thereby enhancing system simplicity, robustness, and cost-effectiveness. In terms of the offset detection algorithm, Frequency-Filtered Angular Measurement Algorithm (FFAMA) is proposed, which is both efficient and succinct, necessitating only a single Fourier transform for its computation. The grating prototype, machined using 3D printing technology, underscores the method’s economic viability. Both simulation and experimental outcomes substantiate that this method achieves detection accuracy at the level of 0.4 arcseconds, with a dynamic range surpassing 1400 arcseconds.

## 2. Theory

### 2.1. The Principle of Talbot Effect

Our offset detection utilizes the self-imaging of a striped amplitude grating at a specific distance, a phenomenon known as the Talbot effect. This phenomenon, discovered in the 19th century [17], has been widely applied in fields such as optical detection and beam shaping. The Talbot effect can be explained in the frequency domain. In the angular spectrum method, the output field $u_{o}$ can be represented in terms of $u_{i}$ as

(1) $$\begin{array}{c} u_{o}\left(x\right)=u_{i}\left(x\right)*h\left(x\right)=IFT\left\{FT\left\{u_{i}\left(x\right)\right\}\cdot FT\left\{h\left(x\right)\right\}\right\} \end{array}$$

where * denotes convolution, h(x) is the propagation kernel, and the angular spectrum method represents the transfer function as

(2) $$\begin{array}{c} H\left(f_{x}\right)=FT\left\{h\left(x\right)\right\}=\exp\left(ikz\sqrt{1-{\left(\lambda f_{x}\right)}^{2}}\right) \end{array}$$

The concept of relative phase $\phi_{rel}\left(f_{x},z\right)$ is introduced to represent the relative phase of any spatial frequency $f_{x}$ with respect to the zero-order diffracted wave [18]:

(3) $$\begin{array}{c} \phi_{rel}\left(f_{x},z\right)=\frac{2\pi z}{\lambda }\left[1-\sqrt{1-{\left(\lambda f_{x}\right)}^{2}}\right] \end{array}$$

When the distance satisfies certain conditions such that the relative phase is an integer multiple of π, periodic images are generated. This distance is referred to as the Talbot distance, $z_{t}$, and the condition that needs to be satisfied by $z_{t}$ is

(4) $$\begin{array}{c} z_{t}=\frac{\lambda }{2[1-\sqrt{1-{\left(\lambda f_{x}\right)}^{2}}]} \end{array}$$

The above equations describe the case of a plane wave vertically incident. When a plane wave is obliquely incident on the grating, there is an offset $x_{0}$ between the light source position and the observation screen position. The expression for the output light field is

(5) $$\begin{array}{cc} u_{o}\left(\hat{x}\right) & =u_{o}^{\prime }\left(\hat{x}+x_{0}\right)\\ \; & =IFT\{FT\left\{u_{i}\left(x\right)\right\}\cdot FT\left\{h\left(\hat{x}+x_{0}\right)\right\}\}\\ \; & =IFT\{U\left(f_{x}\right)H_{n}\left(f_{x}\right)\}\; \end{array}$$

The transfer function can be calculated as

(6) $$\begin{array}{c} H_{n}\left(f_{x}\right)=\exp\left[ik\left(\lambda f_{x}x_{0}\right)+z\sqrt{\left(1-{\left(\lambda f_{x}\right)}^{2}\right)}\right] \end{array}$$

According to the displacement property of the Fourier transform,

(7) $$\begin{array}{c} F\left[f\left(t-t_{0}\right)\right]=e^{-i\omega t_{0}}F\left(\omega \right) \end{array}$$

in the spatial domain, moving by $Z_{t}\tan\theta$.

**Figure 1:**
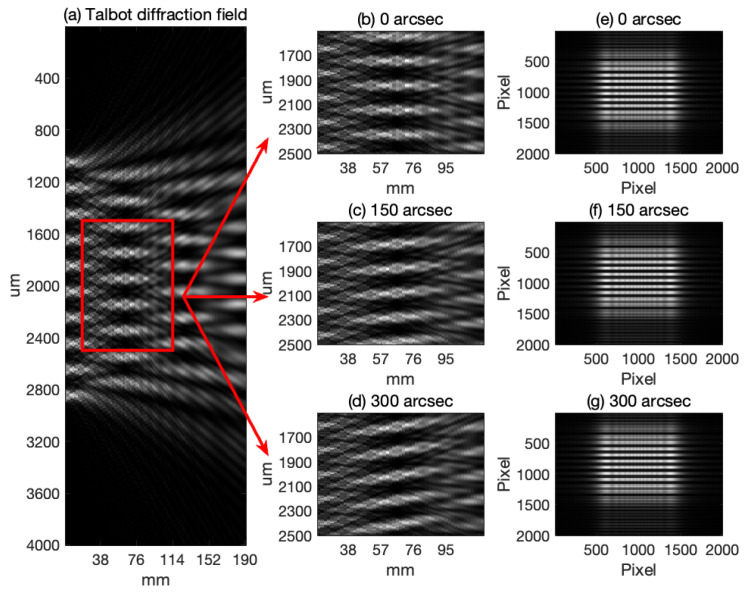
Simulation of diffraction patterns at various incident angles.

Figure 1, derived from simulations utilizing the angular spectrum method, illustrates the diffraction patterns resulting from a plane wave’s interaction with a grating at different angles of incidence. Reference to the Shifted Band-Extended Angular Spectrum Method (SBEASM) [19] ensures the accuracy of the off-axis diffraction calculation in the angular spectrum simulation, providing a solid foundation for our analysis. The grating, characterized by a period of 200 µm, and the wavelength of the plane wave at 632 nm are fundamental parameters in these simulations. Figure 1a presents the overall diffraction field outcome when the plane wave is normally incident on the grating. This figure demonstrates the formation of periodic self-images at the Talbot distance, a phenomenon intrinsic to the Talbot effect. Figure 1b–d are magnified views of the diffraction field when the plane wave is incident on the grating at angles of 0 arcseconds, 150 arcseconds, and 300 arcseconds, respectively. A clear shift in the diffraction pattern is observable with increasing angles of incidence, indicating a dynamic response of the system to angular deviations. Figure 1e–g correspond to the focal plane images of Figure 1b–d, respectively, at the Talbot distance. These figures showcase the self-images of the grating on the focal plane, which exhibit noticeable displacements. By measuring these displacements, we can accurately calculate the change in the angle of incidence. The series from Figure 1b–d, and correspondingly from Figure 1e–g, not only visualizes the effect of angular misalignment on the diffraction pattern but also highlights the method’s potential for precise angular measurement applications.

### 2.2. Principles of Angle Detection and Phase Extraction Algorithms

**Figure 2:**
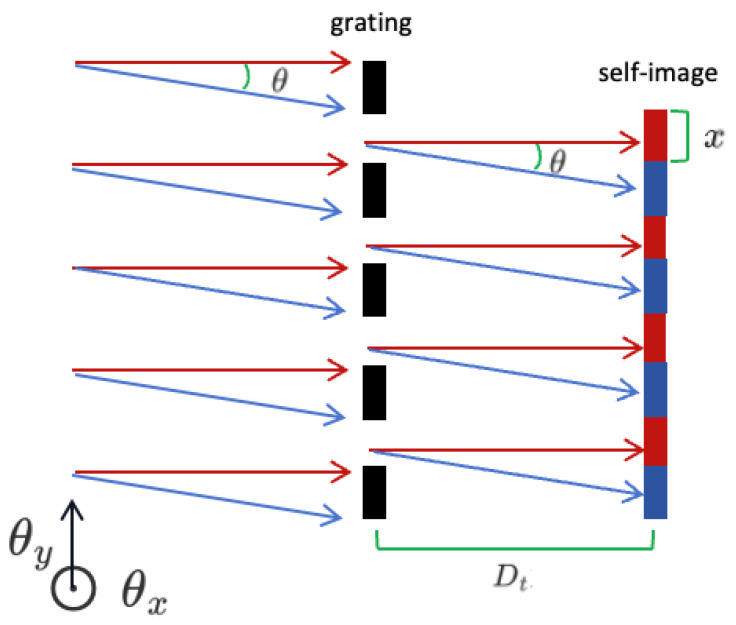
Detection principle schematic diagram (The red arrows denote light incidenting perpendicularly on the grating, while the blue arrows depict light incidenting at an angle θ).

Figure 2 elucidates the mechanism by which our system employs a single grating to measure angular offsets. In the schematic diagram, the direction parallel to the grating lines is defined as the $\theta_{x}$ direction, and the direction perpendicular to the grating lines is defined as the $\theta_{y}$ direction. Central to this process is the Talbot distance ($D_{t}$), the specific location where a plane wave, after interacting with the grating, forms a self-image. When the incident angle of the plane wave is altered by θ, it precipitates a commensurate displacement *x* of the self-image at the focal plane. The measurement of this displacement *x* allows us to accurately calculate the angular offset θ. This relationship can be mathematically expressed as follows:

(8) $$\begin{array}{c} x=D_{t}\cdot \tan\left(\theta \right) \end{array}$$

The angle θ can be expressed as

(9) $$\begin{array}{c} \theta =\tan^{-1}\left(\frac{x}{D_{t}}\right) \end{array}$$

According to the properties of frequency domain transformations, a spatial shift is equivalent to a change in the phase of the frequency domain, and the shift *x* can be extracted from the phase in the frequency domain. Let the spatial domain image be f(x), and after a shift of $x_{0}$, it is represented as $f(x+x_{0})$. The corresponding relationship after undergoing a Fast Fourier Transform (FFT) is as follows:

(10) $$\begin{array}{c} f\left(x\right)\overset{F}{⟷}F\left(\omega \right) \end{array}$$

(11) $$\begin{array}{c} f\left(x+x_{0}\right)\overset{F}{⟷}F\left(\omega \right)\cdot e^{j\omega x_{0}} \end{array}$$

where $\omega =\frac{2\pi n}{N}$, *N* is the width of the image in pixels, and *n* is the pixel position. Dividing the two, the phase information can be expressed as

(12) $$\begin{array}{c} \phi \left(n\right)=angle\left(e^{j\omega x_{0}}\right) \end{array}$$

Let the phase slope be represented as $k_{\phi }$, and by substituting, we can find

(13) $$\begin{array}{c} k_{\phi }=\frac{2\pi x_{0}}{N} \end{array}$$

Substituting into the above equation gives us

(14) $$\begin{array}{c} x_{0}=\frac{k_{\phi }N}{2\pi } \end{array}$$

(15) $$\begin{array}{c} \theta =\tan^{-1}\left(\frac{k_{\phi }N}{2\pi D_{t}}\right) \end{array}$$

Angular changes in the other direction can be calculated using the same method.

Algorithm 1 outlined in our pseudocode streamlines the process of angular offset measurement by incorporating a summation process that lightens the computational load for frequency domain transformations. This summation operation is due to the grating self-imaging distribution pattern, where two-dimensional information is compressed into one dimension without loss of shift information, thereby enhancing computational efficiency and even exhibiting noise reduction effects. Additionally, because the frequency domain characteristics of grating self-images are distinct, extracting information from specific frequency locations can effectively reduce the impact of noise and improve computational accuracy. The phase information, derived from the Fourier transforms of the images, is used to calculate the angular offsets $\theta_{x}$ and $\theta_{y}$ with precision. This method ensures efficient and reliable angular measurements, making it well-suited for high-precision applications.
**Algorithm 1** Frequency-Filtered Angular Measurement Algorithm**Input:** Reference image $I_{\mathrm{ref}}$, displaced image $I_{\mathrm{disp}}$, Talbot distance $D_{\mathrm{Talbot}}$, number of pixels *N*.**Output:** Angular offset in the x-direction $\theta_{x}$; Angular offset in the y-direction $\theta_{y}$.1:Acquire the reference image $I_{\mathrm{ref}}$ and the displaced image $I_{\mathrm{disp}}$.2:Compute the horizontal and vertical sums of $I_{\mathrm{ref}}$ to obtain $S_{\mathrm{row},\mathrm{ref}}$ and $S_{\mathrm{col},\mathrm{ref}}$, respectively. Similarly, compute the horizontal and vertical sums of $I_{\mathrm{disp}}$ to obtain $S_{\mathrm{row},\mathrm{disp}}$ and $S_{\mathrm{col},\mathrm{disp}}$.3:Perform Fourier transform on $S_{\mathrm{row},\mathrm{ref}}$, $S_{\mathrm{col},\mathrm{ref}}$, $S_{\mathrm{row},\mathrm{disp}}$, and $S_{\mathrm{col},\mathrm{disp}}$ to obtain their respective frequency domain representations ${\mathrm{FT}}_{\mathrm{row},\mathrm{ref}}$, ${\mathrm{FT}}_{\mathrm{col},\mathrm{ref}}$, ${\mathrm{FT}}_{\mathrm{row},\mathrm{disp}}$, and ${\mathrm{FT}}_{\mathrm{col},\mathrm{disp}}$.4:Calculate the frequency domain phase information by computing $\phi_{x}=\mathrm{angle}\left({\mathrm{FT}}_{\mathrm{row},\mathrm{disp}}/{\mathrm{FT}}_{\mathrm{row},\mathrm{ref}}\right)$ for the horizontal direction and $\phi_{y}=\mathrm{angle}\left({\mathrm{FT}}_{\mathrm{col},\mathrm{disp}}/{\mathrm{FT}}_{\mathrm{col},\mathrm{ref}}\right)$ for the vertical direction.5:Compute the frequency domain phase slopes $k_{x}=\phi_{x}\cdot {\mathrm{FT}}_{\mathrm{row},\mathrm{ref}}$ in the x-direction and $k_{y}=\phi_{y}\cdot {\mathrm{FT}}_{\mathrm{col},\mathrm{ref}}$ in the y-direction.6:Calculate the angular offsets $\theta_{x}$ and $\theta_{y}$ in the x-direction and y-direction, respectively, using the following formulas:
$$\theta_{x}=\tan^{-1}\left(\frac{k_{x}\cdot N}{2\pi D_{Talbot}}\right),\;\theta_{y}=\tan^{-1}\left(\frac{k_{y}\cdot N}{2\pi D_{Talbot}}\right)$$

## 3. Simulation and Performance Analysis

We conducted simulations of the diffraction process using the angular spectrum method to analyze how a plane wave at varying angles interacts with a grating. The parameters for the simulation are detailed below:

- Wavelength: 632 nm.
- Grating period: 200 µm.
- Focal plane distance: 63.3 mm.
- Pixel size: 2.4 µm.
- Image resolution: 2001 × 2001 pixels.

The simulation encompassed three sets of data, with each set comprising one hundred images. The displacements in each set were specified to be 0.02 pixels, 0.2 pixels, and 2 pixels, corresponding to dynamic ranges of 2 pixels, 20 pixels, and 200 pixels, respectively. These displacements correspond to angular offsets of 0.13 arcseconds, 1.3 arcseconds, and 13 arcseconds, with associated dynamic ranges of 13 arcseconds, 130 arcseconds, and 1300 arcseconds.

### 3.1. Accuracy and Dynamic Range Analysis

**Figure 3:**
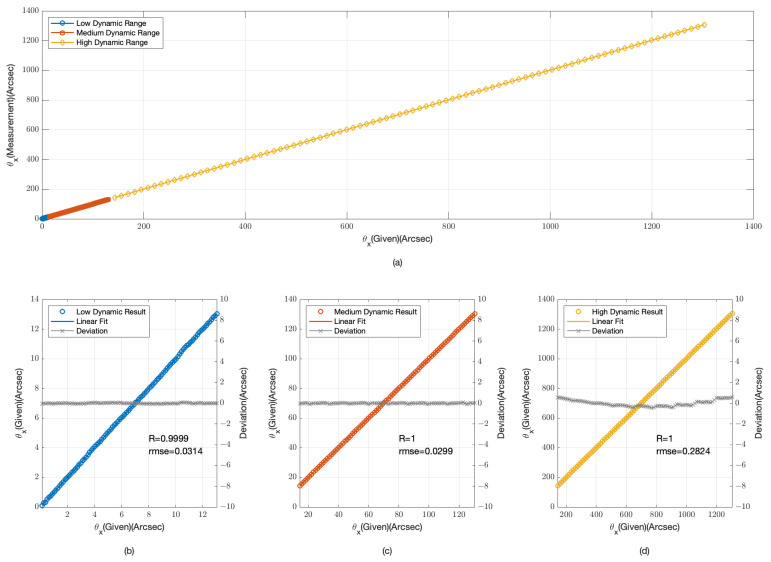
Simulation results for $\theta_{x}$ angular displacement measurements (**a**) overall range; (**b**) low dynamic range; (**c**) medium dynamic range; (**d**) high dynamic range.

**Figure 4:**
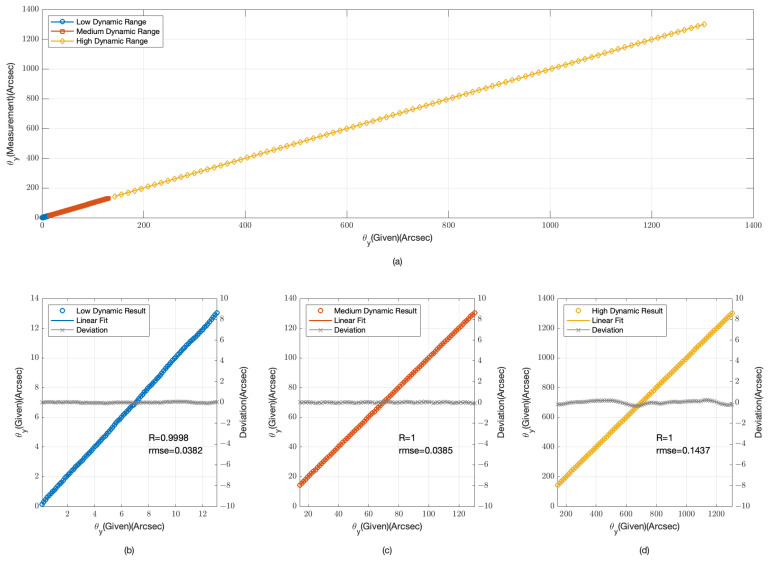
Simulation Results for $\theta_{y}$ angular displacement measurements (**a**) overall range; (**b**) low dynamic range; (**c**) medium dynamic range; (**d**) high dynamic range.

The provided Figure 3 and Figure 4 offer a detailed examination of our angular measurement system’s performance across low, medium, and high dynamic ranges. Figure 3a and Figure 4a compile data from all three dynamic ranges, providing an integrated view of the system’s response to a spectrum of angular offsets.

In the low dynamic range, as depicted in Figure 3b and Figure 4b, the data points are tightly clustered around the linear fit lines, indicating a high degree of accuracy. The root mean square error (RMSE) for the $\theta_{x}$ data is 0.0314 arcseconds with a correlation coefficient (R) of 0.9999, while for the $\theta_{y}$ data, the RMSE is 0.0382 arcseconds and R is 0.9998. These values confirm the system’s high sensitivity and precision for minor angular changes.

For the medium dynamic range, shown in Figure 3c and Figure 4c, the system’s performance remains robust. The RMSE for the $\theta_{x}$ data is 0.0299 arcseconds with R = 1, and for the $\theta_{y}$ data, the RMSE is 0.0385 arcseconds also with R = 1. The slight increase in RMSE compared with the low dynamic range is negligible, suggesting that the system maintains its accuracy as the dynamic range increases.

In the high dynamic range, illustrated in Figure 3d and Figure 4d, the system’s performance is consistent with the previous ranges. The RMSE for the $\theta_{x}$ data is 0.2824 arcseconds with R = 1, and for the $\theta_{y}$ data, the RMSE is 0.1437 arcseconds with R = 1. Although the RMSE is higher in this range due to the larger angular offsets, the system still demonstrates a strong linear relationship, indicating its capability to handle significant angular deviations.

The simulation results from Figure 3 and Figure 4 demonstrate the angular measurement system’s effectiveness across various dynamic ranges. The low RMSE values and high correlation coefficients across all ranges confirm the system’s reliability and accuracy. The system’s performance is particularly noteworthy in the high dynamic range, where it continues to provide accurate measurements despite the challenges associated with larger offsets.

### 3.2. Signal-to-Noise Ratio (SNR) Analysis

**Figure 5:**
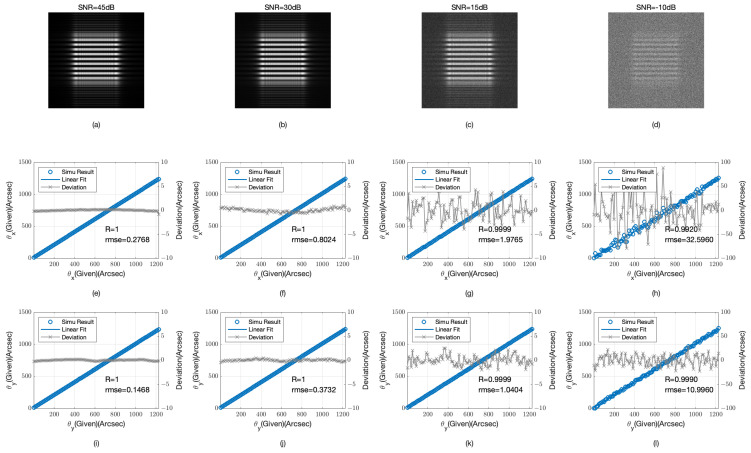
Detection results at varying SNR levels (**a**) image at 45 dB SNR; (**b**) image at 30 dB SNR; (**c**) image at 15 dB SNR; (**d**) image at −10 dB SNR; (**e**) detection results of $\theta_{x}$ at 45 dB SNR; (**f**) detection results of $\theta_{x}$ at 30 dB SNR; (**g**) detection results of $\theta_{x}$ at 15 dB SNR; (**h**) detection results of $\theta_{x}$ at −10 dB SNR; (**i**) detection results of $\theta_{y}$ at 45 dB SNR; (**j**) detection results of $\theta_{y}$ at 30 dB SNR; (**k**) detection results of $\theta_{y}$ at 15 dB SNR; (**l**) detection results of $\theta_{y}$ at −10 dB SNR.

In actual working conditions, noise has a significant impact on detection results. This section will simulate the impact of different signal-to-noise ratio (SNR) conditions on detection outcomes. We chose common Gaussian noise as interference to verify the detection accuracy of the algorithm under varying SNR conditions, with the SNR range set from 45 dB to −10 dB and simulation parameters consistent with those previously described. Figure 5 displays the images and detection results under varying SNR conditions.

At a high SNR of 45 dB, the detection accuracy for both $\theta_{x}$ and $\theta_{y}$ is high, with RMSE values of 0.2766 arcseconds and 0.1468 arcseconds, respectively, and correlation coefficients R close to 1, indicating a very high linear relationship. As the SNR decreases, detection accuracy begins to be affected. At 30 dB, the RMSE for $\theta_{x}$ increases to 0.8024 arcseconds, while for $\theta_{y}$ it is 0.3732 arcseconds. Despite a decrease in accuracy, the correlation coefficient R remains at 1, indicating that the system still maintains a strong linear response. When the SNR drops to 15 dB, the RMSE for $\theta_{x}$ and $\theta_{y}$ increases to 1.9765 arcseconds and 1.0404 arcseconds, respectively, with correlation coefficients R slightly dropping to 0.9999, suggesting that the system can still maintain high detection accuracy under medium SNR conditions. However, under very low SNR conditions, such as −10 dB, detection accuracy significantly declines, with the RMSE for $\theta_{x}$ soaring to 32.5960 arcseconds and for $\theta_{y}$ to 10.9960 arcseconds, and correlation coefficients R dropping to 0.9920 and 0.9990, respectively, indicating that system performance is severely affected in high-noise environments.

These data indicate that as noise increases, the RMSE for both directions increases, especially when the SNR is below 30 dB, where the increase in RMSE is more pronounced. When the SNR approaches 30 dB, the RMSE for the $\theta_{y}$ direction remains below 0.4 arcseconds, illustrating the system’s high detection accuracy in this orientation. However, when the SNR decreases to 15 dB, the RMSE for the $\theta_{x}$ increases to approximately 2 arcseconds. Despite this increase, the linear correlation coefficient remains close to 1, indicating that the system retains a strong linear relationship even in the presence of higher noise levels. This suggests that the algorithm retains a degree of noise resistance, particularly in the $\theta_{y}$ detection results, which are aligned perpendicularly to the grating direction and exhibit stronger resistance to noise.

**Figure 6:**
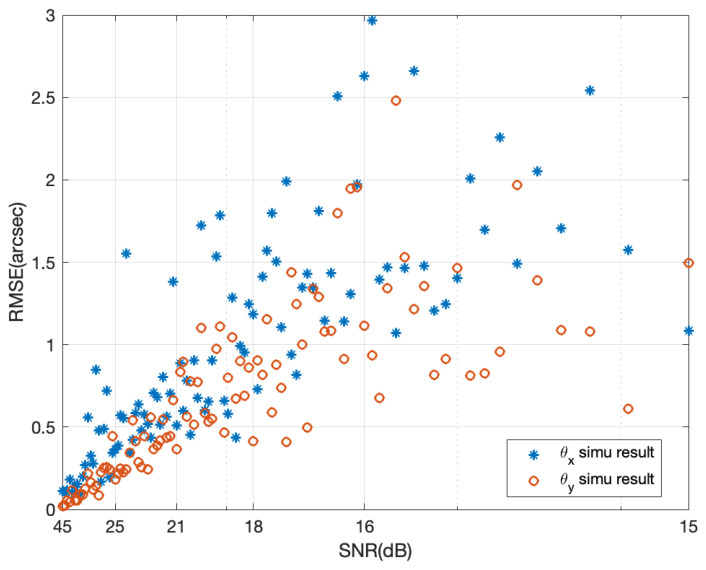
Detection accuracy variation with signal-to-noise ratio (SNR).

Figure 6 shows the variation of $\theta_{x}$ and $\theta_{y}$ as the SNR decreases from 45 dB to 15 dB, with the SNR on the horizontal axis represented on a logarithmic scale. It can be seen from the figure that the detection RMSE continuously increases with the addition of noise, and the variation trend for $\theta_{y}$ is smaller than that for $\theta_{x}$, indicating that the vertical change direction of the grating can extract more frequency domain information and is therefore more resistant to noise. When the SNR is greater than 25 dB, the detection accuracy can basically achieve the 0.4 arcseconds proposed in this paper.

### 3.3. Light Source Aberration Analysis

**Figure 7:**
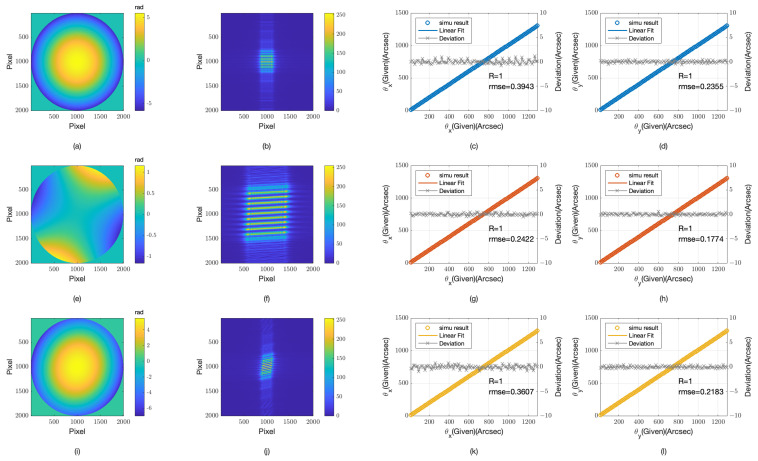
Imaging and detection results under aberrant light sources: defocus, astigmatism, and combined defocus-astigmatism (**a**) defocus aberration; (**b**) grating self-imaging under defocus aberration; (**c**) detection results in the $\theta_{x}$ direction under defocus aberration; (**d**) detection results in the $\theta_{y}$ direction under defocus aberration; (**e**) astigmatism aberration; (**f**) grating self-imaging under astigmatism aberration; (**g**) detection results in the $\theta_{x}$ direction under astigmatism aberration; (**h**) detection results in the $\theta_{y}$ direction under astigmatism aberration; (**i**) combined defocus and astigmatism aberration; (**j**) grating self-imaging under combined defocus and astigmatism aberration; (**k**) detection results in the $\theta_{x}$ direction under combined defocus and astigmatism aberration; (**l**) detection results in the $\theta_{y}$ direction under combined defocus and astigmatism aberration.

The work presented earlier in this study assumes an incident beam in the form of an ideal plane wave, which is challenging to replicate under real experimental conditions. To address this, we have simulated the impact of optical aberrations on the light source using Zernike polynomials. The simulation results are presented in Figure 7. Specifically, we have introduced defocus Figure 7a, astigmatism Figure 7e, and a combination of defocus and astigmatism Figure 7i aberrations, while keeping all other simulation parameters consistent with the aforementioned experiments.

From the focal image (at the Talbot distance), the self-images formed under various aberrated light sources show noticeable changes when compared with those formed by an ideal plane wave. For instance, the defocused self-image at a Talbot distance shows a noticeable reduction in size, as evidenced by the pixel intensity distribution in Figure 7b. Astigmatic illumination results in a certain torsion of the self-image, visible in Figure 7f, while the combination of defocus and astigmatism exhibits characteristics of both, as depicted in Figure 7j. Despite these variations, the self-images, under different offsets, exhibit minimal changes beyond spatial position alterations.

The simulation results indicate that the RMSE increases significantly with the introduction of defocus aberration. For example, the RMSE in the $\theta_{x}$ direction for the defocused condition is 0.3943 arcseconds, a substantial increase compared with the ideal plane wave scenario. This increase is likely due to the reduction in self-image size, which leads to the loss of high-frequency information. In contrast, astigmatism does not have as substantial an impact on the RMSE, with an RMSE of 0.2422 arcseconds in the $\theta_{x}$ direction. This suggests that while astigmatism affects the self-image through torsion, it does not significantly contribute to the loss of high-frequency details that affect precision.

Furthermore, observations indicate that the performance in the $\theta_{y}$ direction is superior to that in the $\theta_{x}$ direction. The simulation results indicate that under astigmatic conditions, the $\theta_{y}$ direction records a lower RMSE of 0.1774 arcseconds, whereas the RMSE for the $\theta_{x}$ direction is 0.2422 arcseconds. This suggests that the $\theta_{y}$ direction retains more information and thus offers better performance, possibly due to the orientation being less susceptible to the introduced aberrations.

## 4. Experimental Preparation and Analysis

**Figure 8:**
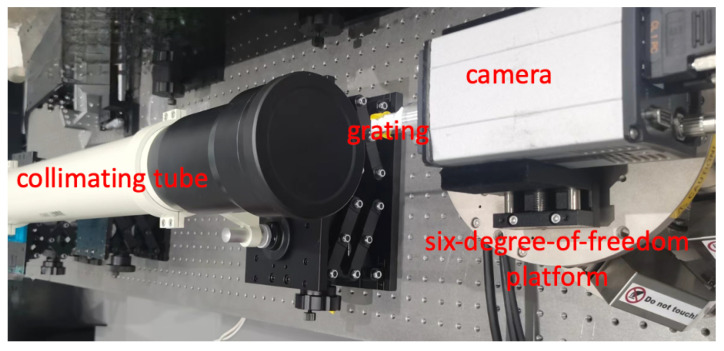
Optical path for angular measurement in physical experiments.

The experimental optical path is depicted in Figure 8, where a point light source generates a plane wave after passing through a collimating tube. After propagating through the grating structure, it travels a specific distance to reach the camera’s focal plane. The camera is mounted on a six-degree-of-freedom platform, which is used to create the angles necessary for measurement. The angular displacement produced by the six-degree-of-freedom platform is calculated based on the shift of the grating image at the focal plane.

The light source in our system offers a stable output wavelength of 632 nm and an output power of 5 mW. During the alignment procedure, adjustments are meticulously made to the emitted circular light to maintain uniform diameters at both proximal and distal ends, thereby generating an approximate collimated beam. These refinements ensure the laser beam’s propagation as a plane wave with minimal divergence.

The six-degree-of-freedom platform, model PI H-824, is meticulously calibrated with an interferometer to ensure the accurate generation of angular displacements. This precision is paramount for our experiments. The platform’s linearity, rated at better than 0.01%, is crucial for preserving the accuracy and reliability of our angular measurements.

The diffraction grating, central to our experimental path, is crafted using 3D printing technology. This grating, characterized by an accuracy of 10 µm, a period of 200 µm, and dimensions of 5 mm, has been designed with two sets of gratings oriented perpendicularly to each other. This design, informed by simulation results indicating higher detection precision perpendicular to the grating lines, ensures precision measurement in both the $\theta_{x}$ and $\theta_{y}$ directions. Manufactured by the nanoArch S140, which is a micro-nano 3D printer developed by BMF Precision Tech Inc. from Shenzhen City, Guangdong Province, China, this grating is characterized by an accuracy of 10 µm, a period of 200 µm, and dimensions of 5 mm. The detection of diffracted light is facilitated by a high-resolution QHY183M detector, which is manufactured by QHYCCD, a company based in Beijing, China. Characterized by a pixel size of 2.4 µm and a substantial pixel scale of 5544 × 3694, this detector provides the fine resolution required to capture the subtleties of the diffraction pattern with clarity. Its integration with the grating is meticulously aligned to ensure precise data acquisition for our experiments.

**Figure 9:**
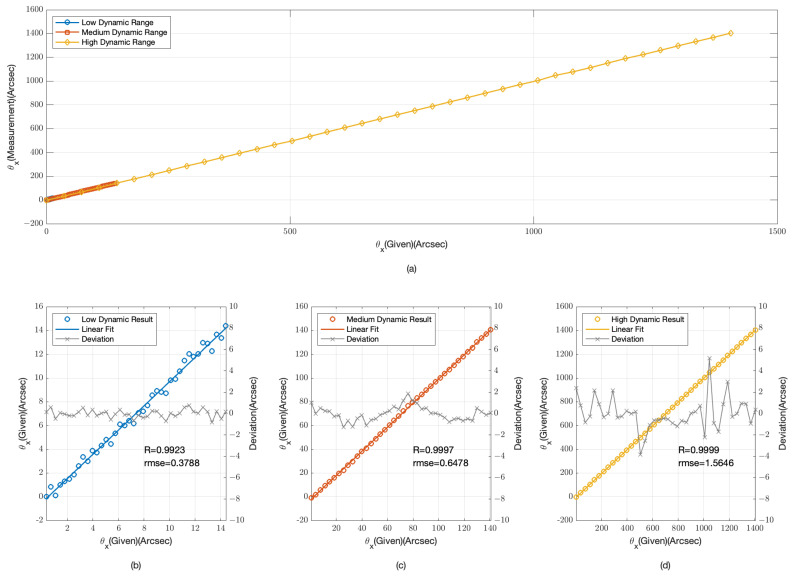
Experimental results for $\theta_{x}$ angular displacement measurements (**a**) overall range; (**b**) low dynamic range; (**c**) medium dynamic range; (**d**) high dynamic range.

**Figure 10:**
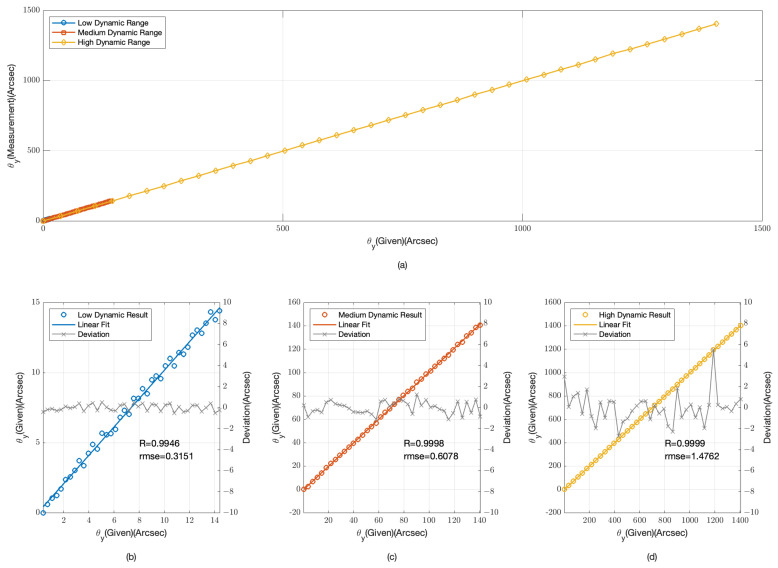
Experimental results for $\theta_{y}$ angular displacement measurements (**a**) overall range; (**b**) low dynamic range; (**c**) medium dynamic range; (**d**) high dynamic range.

The physical experiments, analogous to the simulation depicted in Figure 3 and Figure 4, were conducted to assess the angular measurement system’s performance across low, medium, and high dynamic ranges. The experimental results, as illustrated in Figure 9 and Figure 10, mirror the simulated data, offering a practical validation of the system’s response to a variety of angular offsets.

In the low dynamic range, the physical experimental results align with the simulation, demonstrating a high degree of accuracy. The data points closely follow the linear fit lines, with an RMSE of 0.3788 arcseconds for $\theta_{x}$ and 0.3151 arcseconds for $\theta_{y}$. The correlation coefficients are notably high at R = 0.9923 and R = 0.9946, respectively. These results underscore the system’s sensitivity and precision in measuring minor angular changes.

For the medium dynamic range, the physical experiments exhibit a sustained level of performance. The RMSE values are 0.6478 arcseconds for $\theta_{x}$ and 0.6078 arcseconds for $\theta_{y}$, with correlation coefficients of R = 0.9997 and R = 0.9998. The consistency in performance despite the increased dynamic range reaffirms the system’s ability to maintain accuracy.

In the high dynamic range, the physical experiments continue to demonstrate the system’s robustness. The RMSE values are 1.5646 arcseconds for $\theta_{x}$ and 1.4762 arcseconds for $\theta_{y}$, accompanied by correlation coefficients of R = 0.9999 and R = 0.9999. Although the RMSE is higher due to the larger angular offsets, the strong linear relationship indicates the system’s capability to manage significant angular deviations effectively.

The physical experimental results, while affirming the general performance trends observed in the simulations, did not achieve the same high degree of precision. This deviation could be attributed to several factors inherent in real-world experiments. Firstly, the detection optical path in our experiment is mounted on a pneumatic isolation platform, which effectively minimizes the influence of most environmental vibrations. Despite this, the relative positional relationships among the collimating tube, camera, and six-degree-of-freedom platform could potentially introduce deviations. To address this, future experiments should integrate higher-precision collimators or interferometers for synchronized measurements, ensuring the accuracy of the angular deviations captured. Additionally, the thickness of the grating, which is 0.5 mm in this study, introduces an edge effect when light is incident at an angle. This results in a deviation of approximately 3 μm at 1400 arcseconds, affecting the precision of angle extraction. Furthermore, as the angle increases, phase aliasing in the frequency domain becomes more pronounced, naturally leading to greater extraction errors. Lastly, noise from the detector can introduce uncertainties into the measurements, further impacting the overall accuracy of the system. However, compared with the simulation results, by incorporating two sets of gratings that are perpendicular to each other, the detection precision in both the $\theta_{x}$ and $\theta_{y}$ directions no longer exhibits a significant difference.

**Figure 11:**
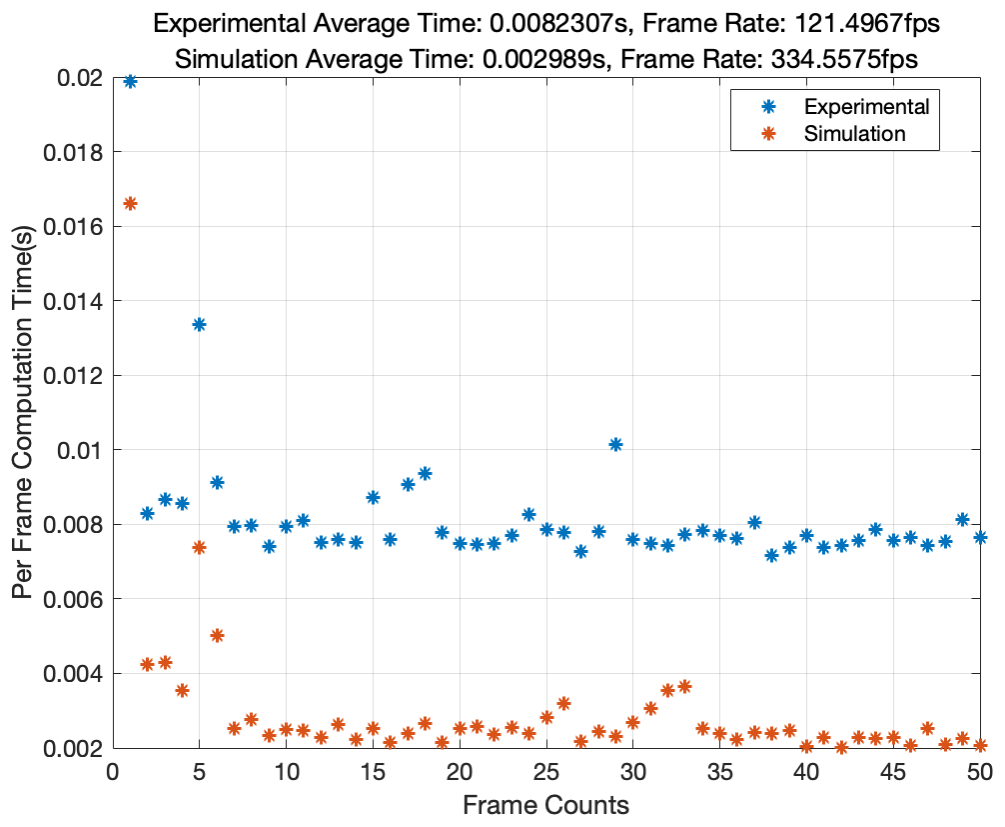
Algorithm performance analysis: simulation vs. experimental data.

Figure 11 presents a comprehensive analysis of our algorithm’s time efficiency and highlighting its suitability for real-time processing applications. The algorithm exhibits remarkable performance in both simulated and experimental scenarios. In simulations, it achieves an average computation time of 0.002989 s per frame, equating to a high frame rate of 334.5575 fps. In contrast, the experimental data, which involve processing a higher number of pixels and larger per-frame data volumes, demonstrate a computation time of 0.008231 s per frame, resulting in a frame rate of 121.4967 fps. While the experimental frame rate is slightly lower due to the increased pixel count and data volume per frame, it still underscores the algorithm’s capability for rapid data processing.

The analysis was performed using MATLAB R2022a, a platform renowned for its robust computational tools, executed on a system powered by Apple’s M2 chip.

## 5. Discussion

Table 1 presents a comparative analysis of various angular measurement techniques, including our own work and other reported methods in the literature. Each method is categorized based on its underlying technology and assessed for key performance metrics such as dynamic range, accuracy, and required length.

MEMS-based method [20]: Classified as a micro-electromechanical system (MEMS), this method boasts a large dynamic range and a compact form factor. However, its accuracy does not meet the stringent requirements typically demanded by optical systems.

Autocollimator method [8]: Geometrical optics-based, this approach offers a suitable dynamic range and accuracy aligned with optical system specifications. Despite its performance, it suffers from a larger footprint and higher costs due to its complex setup.

Grating-based method [14]: falling under the category of wave optics, this method is highly compact but susceptible to noise interference, which impacts its measurement precision.

Our method: Our proposed technique utilizes a single-layer, 3D-printed amplitude grating. It excels with a high dynamic range and precision, meeting the high standards of optical systems. Additionally, it occupies minimal space and is cost-effective, making it an attractive solution for applications where precision and compactness are paramount.

The method presented in this paper is universal and can be integrated into various systems, including those that utilize holographic diffractive elements or defocused images from arbitrary lenses. However, compared with gratings, these alternatives have certain disadvantages. The grating used in this study is an amplitude-type grating, which can be fabricated using 3D printing technology, whereas lenses or diffractive elements are phase-type elements that require optical fabrication equipment, resulting in significantly different cost levels. Secondly, phase-type elements often introduce different wavefront aberrations across various fields of view, causing frequency domain interference and measurement errors at different positions. In contrast, the Talbot images formed by gratings possess self-healing capabilities [21] and are more tolerant to tolerances. Lastly, the Talbot diffraction field has multiple Talbot distances, enabling the adjustment of dynamic range and precision by selecting appropriate working distances.

The method presented in this study offers significant advantages in terms of miniaturization. The grating-based detection optical path requires only about 6 cm in length, and when combined with a compact camera, it is possible to achieve a detection system within the 10 cm scale. Our analysis of optical aberrations revealed that the quality of collimated light has a minimal impact on detection accuracy. Moreover, the small size of the grating allows for the use of a laser in conjunction with a single lens to generate an approximate collimated beam. Given the compact nature of the entire setup, it can be rapidly and conveniently applied to fields such as rough alignment adjustment of optical systems, posture monitoring, and robotics.

## 6. Conclusions

In this study, we have successfully developed and tested a high-precision angle measurement device that employs the Talbot effect. The device features a simple design, relying on a single grating to achieve a commendable degree of accuracy in angle detection. We have applied a phase extraction technique in the frequency domain, which has proven to be relatively efficient in computation and effective in maintaining accuracy.

By using a six-degree-of-freedom controller to induce angular deflections, our prototype has demonstrated an ability to measure angles over a dynamic range of up to 1400 arcseconds with an accuracy of 0.4 arcseconds. The device also exhibits excellent linearity at 0.03%, offering a reliable solution for precision measurement applications.

Looking forward, our goal is to further refine this technology. We plan to continue enhancing the device’s precision and expanding its dynamic range. Our future work will focus on exploring ways to improve its accuracy even further and on identifying new areas where our device could contribute, such as in enhancing optical alignment processes or stabilizing phases in various precision optical applications.

## Figures and Tables

**Table 1:** Comparison between this work and other reported.

Reference	Year	Range	Accuracy (% FS)	Length (mm)	Type
[20]	2018	360∘	850″ (0.066%)	5.5	Mems
[8]	2020	400″	0.74″ (0.19%)	500	Autocollimator
[14]	2023	800″	80″ (10%)	0.4	Grating-based
Our method	2024	1400″	0.4″ (0.03%)	65	Grating-based

## Data Availability

Data are contained within the article.
